# Cognitive Aging and Dementia Research: Expanding Horizons on Social and Environmental Contexts

**DOI:** 10.1093/geroni/igaf122.1295

**Published:** 2025-12-31

**Authors:** Jacqui Smith, Dawn Carr

## Abstract

This symposium showcases new cross-disciplinary research into proposals about early-life and midlife contexts associated with later-life cognition and dementia. The early-career speakers utilize data from the Carolina African American Twins Study of Aging (CAATSA), Health and Retirement Study (HRS), and Panel Study of Income Dynamics (PSID) and apply advanced analysis methods. Presentations consider the influence of education, early adult and midlife neighborhood poverty, and pre-retirement work contexts in cognitive aging. Early-life education is well-known as a predictor of dementia. Johnson expands this research by revealing a unique role for education beyond zygosity and socio-environmental factors. Kalousová examines the relevance to late life cognition of early-to-late life residential contexts characterized by neighborhood poverty. Using sequence analysis, she identifies distinct patterns of exposure to neighborhood disadvantage over time. Cumulative exposures to areas with limited resources increased the likelihood of late-life cognitive impairment. Retirement from the workforce often contributes to increased stress, loneliness, and reduced cognitive engagement. Kayser, Xiang, and Smith apply cross-wave dynamic change models to HRS multi-wave data to disentangle bidirectional associations between changes in cognition and loneliness after retirement. They report that over the longer-term both cognitive decline and loneliness increase. Working longer and different retirement trajectories may mitigate cognitive decline. Oh used sequence analysis to identify six work-related trajectories prior to retirement. He finds that working longer and engaging in so-called “encore” careers reduce the probability of later-life dementia. The session concludes with an integrative discussion by Carr of the implications of these studies for future research and policy.

